# Inferring Numbers of Wild Poliovirus Excretors Using Quantitative Environmental Surveillance

**DOI:** 10.3390/vaccines9080870

**Published:** 2021-08-06

**Authors:** Yuri Perepliotchikov, Tomer Ziv-Baran, Musa Hindiyeh, Yossi Manor, Danit Sofer, Jacob Moran-Gilad, Laura Stephens, Ella Mendelson, Merav Weil, Ravit Bassal, Emilia Anis, Shepherd Roee Singer, Ehud Kaliner, Gillian Cooper, Manasi Majumdar, Michal Markovich, Daniela Ram, Itamar Grotto, Ronni Gamzu, Javier Martin, Lester M. Shulman

**Affiliations:** 1 Central Virology Laboratory, Sheba Medical Center, Tel Hashomer, Ramat Gan 52621, Israel; yuriper1@gmail.com (Y.P.); mhindiyeh@avh.org (M.H.); y-manor@hotmail.com (Y.M.); danitso@sheba.health.gov.il (D.S.); ellamen@sheba.health.gov.il (E.M.); Merav.Weil@sheba.health.gov.il (M.W.); dr.daniela.ram@gmail.com (D.R.); 2 School of Public Health, Sackler Faculty of Medicine, Tel Aviv University, Tel Aviv 6997801, Israel; zivtome@post.tau.ac.il (T.Z.-B.); Ronni@tlvmc.gov.il (R.G.); 3 Public Health Services, MOH, Jerusalem 9101002, Israel; giladko@post.bgu.ac.il (J.M.-G.); emilia.anis@moh.health.gov.il (E.A.); roee.singer@moh.health.gov.il (S.R.S.); ehud.kaliner@moh.health.gov.il (E.K.); itamar.grotto@moh.health.gov.il (I.G.); 4 Faculty of Health Sciences, Ben Gurion University of the Negev, Beer Sheva 8410501, Israel; 5 National Institute for Biological Standards and Controls, Blanche Lane, South Mimms, Potters Bar, Hertfordshire EN6 3QG, UK; laura.stephens@nibsc.org (L.S.); Gill.Cooper@nibsc.org (G.C.); Manasi.Majumdar@nibsc.org (M.M.); Javier.Martin@nibsc.org (J.M.); 6 Israel Center for Disease Control, Ministry of Health, Gertner Building, Sheba Medical Center, Tel Hashomer, Ramat Gan 52621, Israel; Ravit.Bassal@moh.gov.il (R.B.); michaluch@yahoo.com (M.M.); 7 Braun School of Public Health and Community Medicine, Hebrew University Hadassah Faculty of Medicine, Ein Kerem. P.O. Box 12271, Jerusalem 9112102, Israel

**Keywords:** poliovirus, outbreaks, quantitative environmental surveillance, vaccination, oral poliovirus vaccine, inactivated poliovirus vaccine, asymptomatic infections, stools, sewage, composite sewage samples, vaccine-derived poliovirus

## Abstract

Response to and monitoring of viral outbreaks can be efficiently focused when rapid, quantitative, kinetic information provides the location and the number of infected individuals. Environmental surveillance traditionally provides information on location of populations with contagious, infected individuals since infectious poliovirus is excreted whether infections are asymptomatic or symptomatic. Here, we describe development of rapid (1 week turnaround time, TAT), quantitative RT-PCR of poliovirus RNA extracted directly from concentrated environmental surveillance samples to infer the number of infected individuals excreting poliovirus. The quantitation method was validated using data from vaccination with bivalent oral polio vaccine (bOPV). The method was then applied to infer the weekly number of excreters in a large, sustained, asymptomatic outbreak of wild type 1 poliovirus in Israel (2013) in a population where >90% of the individuals received three doses of inactivated polio vaccine (IPV). Evidence-based intervention strategies were based on the short TAT for direct quantitative detection. Furthermore, a TAT shorter than the duration of poliovirus excretion allowed resampling of infected individuals. Finally, the method documented absence of infections after successful intervention of the asymptomatic outbreak. The methodologies described here can be applied to outbreaks of other excreted viruses such as severe acute respiratory syndrome coronavirus 2 (SARS-CoV-2), where there are (1) significant numbers of asymptomatic infections; (2) long incubation times during which infectious virus is excreted; and (3) limited resources, facilities, and manpower that restrict the number of individuals who can be tested and re-tested.

## 1. Introduction

Three non-cross-reacting serotypes of poliovirus can cause poliomyelitis, also known as polio/Heine-Medin disease/infantile paralysis. Acute flaccid paralysis (AFP) is “a clinical syndrome characterized by rapid onset of weakness, including (less frequently) weakness of the muscles of respiration and swallowing, progressing to maximum severity within several days to weeks” [1]. One cause of AFP is inflammation and destruction of nerve cells in the brain stem or the spinal cord after infection with polioviruses, which results in motor paralysis and atrophy of skeletal muscle [2,3]. While most poliovirus infections are asymptomatic, there is a poliomyelitis case-to-infection ratio for serotypes 1, 2, and 3 infections of 1 to 180, 1 to 1886, and 1 to 1149 infections, respectively, in unvaccinated or under-vaccinated populations [4]. Thus, there may be many infected individuals in a population before the first AFP case occurs. In 1988, the World Health Assembly declared poliomyelitis as the next human disease to be eliminated [5] after successful eradication of smallpox in 1980 [6]. Globally coordinated universal vaccination of all children and poliovirus surveillance were the main means proposed for eliminating polio by this Global Polio Eradication Initiative. Two types of polio vaccines were available, vaccines based on inactivated polio virus (IPV) and vaccines based on attenuated oral polio virus strains (OPV). Both types of vaccines were available in trivalent form, e.g., tIPV and tOPV both contained representative polio vaccine strains for each of the three serotypes of poliovirus, although, in some countries, routine vaccination was based on use of one or more monovalent OPV vaccine strains [7,8,9]. The three main methods of polio surveillance that are used to date are AFP surveillance, enterovirus surveillance, and environmental surveillance (EnvS).

AFP surveillance is the classic method for determining whether poliovirus is circulating in a given population [10]. The incidence of AFP from all causes other than polio is 1 per 100,000 for children under the age of 15. AFP surveillance is designed to detect poliovirus infections by screening two stool samples collected at least 24 h apart within 14 days of paralysis onset [10,11] from all cases of AFP. Poliovirus infections are considered to be absent when the incidence of investigated AFP cases is ≥1 per 100,000 children and stool samples from all of the AFP cases are poliovirus-negative. However, the ratio of symptomatic infections to asymptomatic infections decreases significantly when polioviruses circulate in highly vaccinated populations [9,12,13,14]. In such populations, syndrome-based surveillance becomes much less effective for early warning. In countries with 90% vaccine coverage, routine monthly EnvS may be 10^3^ to 10^4^ times more sensitive than AFP surveillance [15] because EnvS detects poliovirus excreted by all infected individuals. For example, in 2013, the same wild type 1 poliovirus strain resulted in 38 cases of poliomyelitis in Syria and Iraq, where conflict disrupted routine immunization programs [16], while there were no cases of poliomyelitis in Israel, where poliovirus transmission lasted almost a year, although >90% of the children received ≥3 doses of tIPV polio vaccine [17].

Israel employed AFP surveillance from the early 1970s. The last cases of poliomyelitis caused by wild poliovirus in Israel occurred in 1988 [17,18]. Routine monthly EnvS was initiated in 1989 [19,20]. EnvS sites were geographically distributed throughout the country, and the catchment populations represented 30–40% of the entire population. This was increased to 80% coverage in 2013 at the height of the circulation of wild type 1 poliovirus in that year [21]. Between 1989 and 2005, routine immunization in Israel employed three doses of IPV at 2, 4, and 12 months plus three doses of tOPV at 4, 6, and 12 months [9,18]. During the interval between 2005 and 2013, EnvS samples in Israel were free from polioviruses that could replicate in cells and cause cytopathic effects (CPE) or produce plaques, except for occasional isolations of highly diverged, vaccine derived polioviruses (VPDVs) from unidentified individuals [20,22] and vaccine-like poliovirus strains in EnvS samples from catchment populations that included Palestinian children vaccinated by the Palestinian Health Authority, which did not discontinue routine immunization with a combination of tIPV and tOPV.

Global use of tOPV, tIPV, or a combination of both reduced the number of countries where wild poliovirus circulated endemically from 125 to two—Afghanistan and Pakistan [23]—and the number of paralytic poliomyelitis cases decreased from over 350,00 per year to 33 globally between 1988 and 2018 [24]. Poliomyelitis caused by wild type 2 was not observed since 1999, while poliomyelitis caused by type three was not reported after November 2012 [25]. On 20 September 2015, the Global Commission for the Certification of Poliomyelitis Eradication (GCC) concluded that wild poliovirus type 2 (WPV2) was eradicated worldwide [23,26]. This was followed in April of 2016 by a coordinated global shift away from use of tOPV to bivalent OPV (bOPV, an oral polio vaccine that contains only OPV serotypes 1 and 3) by removing the type 2 vaccine strain component of tOPV. On 1 October 2019, the Global Certification Committee declared that wild type 3 poliovirus was eradicated [27,28], however, type 3 OPV is not yet withdrawn from the oral polio vaccine. In 2014, the WHO Director General declared the international spread of poliovirus as a Public Health Emergency of International Concern (PEHIC) that is still in effect [29]. There is a need to rapidly detect: (1) poliovirus introduction in already polio free countries [29], (2) newly established outbreaks of vaccine-derived polioviruses (VDPVs) resulting from use of monovalent OPVs in supplementary immunization programs in response to a cVDPV outbreak [30], and (3) introduction of cVDPVs into countries where wild poliovirus circulation was not yet interrupted [29].

Israel switched from a routine vaccination schedule using a combination of three doses each of IPV and tOPV to exclusive use of IPV in 2005 [18]. In response to the introduction and the subsequent circulation of wild type 1 poliovirus (WPV1-SoAS) in 2013 [17,21], all children under ten years old were vaccinated starting on 8 August 2013 with a single round of bOPV in a nationwide vaccine campaign; children in the epicenter of the circulation in the Southern Health District were vaccinated with a second dose of bOPV, and two doses bOPV were reintroduced in the routine childhood vaccination program from January 2015. Vaccination histories of all Israeli children are kept on an electronic database.

Knowledge gained from studies over the years on poliovirus infections and surveillance methodology in Israel by the Central Virology Laboratory were incorporated into the model we present here for inferring the number of wild poliovirus infections in a population by quantitative analysis of poliovirus RNA extracted directly from EnvS samples. This model based on direct quantitative measurement of poliovirus RNA in environmental surveillance significantly extends the range of epidemiological information provided by classical EnvS and is especially suited for control of poliovirus outbreaks. The unique combination of studies included: (1) determining the average titers of virus excreted per gram of stool for different OPV strains in Israeli children who were immunized against poliovirus; (2) determining the number of these children who excreted the OPV strains, the length of time that the children continued to excrete vaccine strains, and how much virus they excreted; (3) correlating quantitative vaccine recovery from EnvS samples with information on the number of individuals in the catchment areas vaccinated and the dates that they were vaccinated; (4) determining the correlation between quantitative molecular assay results for poliovirus RNA and different quantitative tissue culture assays for poliovirus viability for polioviruses recovered from sewage; and (5) studying factors that affected quantitative extraction and assay of poliovirus RNA extracted directly from EnvS Samples.

## 2. Materials and Methods

### 2.1. Polioviruses

Wild type 1 poliovirus in Israel that originated in south Asia (WPV1-SoAS) was isolated from stool survey samples collected in 2013 as described in [31]. “Stool survey” refers to the testing of a large number of stool samples from asymptomatic individuals to determine the point prevalence of poliovirus infections in a target population where EnvS but not AFP surveillance demonstrated the presence of high concentrations of poliovirus infected individuals. OPV type 1 poliovirus (OPV1) was isolated from stool survey samples collected after a nation-wide supplementary immunization response targeting all children up to 10 years of age starting on 8 August 2013 [17,21]. Thus, children under 10 years of age were the primary excretors of OPV1.

A weighed scoop of stool was suspended in a measured volume of Ca^+^/Mg^+^-free phosphate buffered saline, PBS^−/−^ (NaCl 8 g, KCl 0.2 gm, Na_2_HPO_4_ 1.15 gm, and KH_2_PO_4_ 0.2 gm, 1 L water; pH = 7.3–7.4) for plaque assay (below). Titers were expressed as the number of plaques calculated per gram of stool. The average daily amount of feces excreted was set to 99.8 g based on a report by Tucker et al. [32], who measured the average amount of feces excreted per day over a 30 day period for adults following a control diet.

Safety tested and approved oral polio virus (OPV) monovalent vaccine bulks types 1 (mOPV1), 2 (mOPV2), and 3 (mOPV3) were kindly provided by an established manufacturer. Virus titers and MAPREC tests were confirmed under NIBSC (Potters Bar, UK) quality systems to standard ISO-17025. The nominal potencies of each stock were 5.12 × 10^8^, 1.02 × 10^8^, and 5.01 × 10^8^ TCID50 per ml, respectively. The bulk stocks were thawed, divided into aliquots of 50 mL and 1 mL, and stored at −70 °C until use. Titers in plaque forming units per milliliter (PFU/mL) were determined by plaque assay [33] at the Central Virology Laboratory (Tel Hashomer, Israel). The three monovalent poliovirus bulk stocks contained 1.3 × 10^8^, 7.4 × 10^7^, and 1.3 × 10^8^ PFU/mL, for types 1, 2, and 3, respectively.

### 2.2. Polio Vaccination Status in Israel at the Times Studies Were Performed

IPV was used exclusively for routine immunization when OPV monovalent strains were introduced into the sewage system for determining quantitative recovery at downstream EnvS sites (Section 2.3.3, below). The IPV was administered at 2, 4, 6, 12–18 months, and 7 years of age.

Crude vaccine virus excretion rates for children who received a dose of tOPV after being vaccinated with three doses of IPV and three doses of tOPV were taken from our studies conducted prior to 2005 [34]. At the end of each successive week after they received the additional dose of tOPV, 36%, 11.8%, 14%, and 9.5% of the vaccinated individuals excreted OPV1. Quantitation of OPV1 in EnvS and stool samples was conducted during the supplementary immunization with bOPV in 2013. The number of individuals in the catchment population of each EnvS site who were vaccinated with bOPV each week for the four weeks preceding the date of collection of each EnvS was obtained from the National Vaccination Registry. The number of individuals who actively excreted vaccine on the date of collection of an EnvS sample, *NExt_OPV1_est_*, was estimated from the weekly number of vaccinations in the four weeks prior to collection using the crude excretion rates from the 2005 study.

Quantitation of WPV1-SoAS in EnvS and stool samples was performed during 2013 and 2014, starting before the supplementary immunization with bOPV and continuing afterwards.

### 2.3. EnvS Samples

#### 2.3.1. Nomenclature

The names of the EnvS sites in this manuscript for the spiking/recovery experiments refer to specific EnvS sites located along the sewage system in the greater Tel Aviv region of Israel (see map in Supplement Figure S1 or Figure S2). “Shaf Dan” refers to an in-line composite sampler located at the inlet to the Shaf Dan Wastewater Treatment Plant, “Reading” refers to an EnvS site immediately upstream of a pumping station on one of the major trunk lines leading to the wastewater treatment plant, and “Cnumber” refers to specific manholes located along branch C, one of the major trunk lines extending upstream from the Reading pumping station. The higher the number is, the farther the site is from the Reading pumping station. The names of the EnvS sites indicate the names of the sewage treatment plant and should not be confused with specific cities or communities with the same name. EnvS sites located at the mouth of trunk lines located upstream from these sites have -Br included in the EnvS name (example: Arad-Br-Kseife is the name for the EnvS site at the mouth of the Kseife branch of the sewage system leading into the Arad Sewer Treatment Plant).

#### 2.3.2. Collection

EnvS samples were collected using automatic in-line collectors at the mouth of sewage treatment plants or automatic, portable, computerized composite sewage collectors (Sigma SD900 portable samplers, HACH, Loveland, CO, USA) for upstream EnvS sites. The EnvS samples were composite samples, e.g., pools of sewage aliquots collected at 30 min intervals over 24 h as previously described [33]. Exceptions to this protocol such as those for the spiking/recovery experiments were specifically indicated. Catchment populations within each catchment area were obtained from the Israel National Statistics Bureau and municipal engineers in charge of the individual sewage treatment facilities. Samples collected between January 2005 and February 2013 were expected to be poliovirus vaccine free, although EnvS samples from Jerusalem, the greater Tel Aviv area, and Haifa occasionally contained VDPVs and EnvS sites that included Palestinians in their catchment populations and occasionally contained OPV-like isolates. EnvS samples collected between February 2013 and August 2013 potentially contained WPV1-SoAS, those collected between 8 August and April 2014 potentially contained WPV1-SoAS, OPV1, and/or OPV3, while EnvS samples collected after April 2014 potentially contained OPV1 and/or OPV3 [17,21].

#### 2.3.3. Sewage System Spiking/Recovery Experiments

See Supplement Figure S1 or Figure S2 for a Map of the Sewage System and the Spiking and EnvS Sites.

The sewage system spiking/recovery experiments described here were performed in 2011–2012 before WPV2 and WPV3 were declared eradicated [26,27,28,30], tOPV withdrawn and replaced by bOPV [26], and GAP III containment of PV2 initiated [35].

Protocol 1: Aliquots of the three monovalent poliovirus bulk stocks were pooled for initial spiking/recovery experiments. The pooled poliovirus was introduced into the Shaf Dan sewage system serving greater Tel Aviv at a single site; C121 and composite EnvS sewage samples were collected at a number of downstream sites at hourly intervals for up to two days.

Protocol 2: In subsequent experiments, aliquots of the three serotypes were introduced separately at C121 and at one or more public toilets emptying into Branch C of the Shaf Dan sewage system. The serotype introduced into the sewage system at site C121 was dripped into the site over a 30 min period using a peristaltic pump, while toilets were flushed twice. The interval between collections of aliquots by the portable composite samplers was reduced to 6 or 10 min so that at least two to three samples at each downstream collection site potentially contained poliovirus from the spike. A single 24 h composite sample collected at hourly intervals was obtained from the in-line automatic composite sampler located at the inlet into the Shaf Dan wastewater treatment plant. Batteries and collection bottles were swapped out with fresh bottles and batteries for back-to-back, continuous collection runs. The portable automatic sampler at C75 was modified to collect individual, separate, un-pooled samples at 6 min intervals to provide a more detailed time course for recovery.

### 2.4. Tissue Culture

Tissue culture testing for the presence of poliovirus was conducted by World Health Organization recommended methods as described [10,33] using human rhabdosarcoma (RD) cells and transgenic murine cells expressing the CD155 human poliovirus receptor (L20B). These World Health Organization certified cell lines were obtained from the NIBSC (Potters Bar, UK). They were maintained in complete Eagle’s MEM-NAA medium (Eagle’s MEM-NAA medium, 10% (*v*/*v*), fetal bovine serum, and 2 mL (*v*/*v*) per 500 mL of PSMY (penicillin G (50,000 U/mL), dihydro-streptomycin (50 mg per mL), and mycostatin (6250 U/mL) per 500 mL) and 4 mL per 500 mL of 3% (*w*/*v*)L-glutamine in water) or copleteM199 medium (M199 medium, 10% fetal bovine serum, and 24 mL of PMSY/L, respectively.

### 2.5. Viral Assays

#### 2.5.1. Tube Culture Assays

Four replicates of two mL tube cultures of RD and of L20B were used for viral challenge after replacing the respective growth mediums with complete viral growth medium (Eagle’s MEM-NAA 302 medium, 2% FBS (*v*/*v*), 4 mL per 500 mL PSMY). Each tube was challenged with 0.2 mL of concentrated sewage [33]. Cytopathic effects (CPE) were measured after 5 days.

#### 2.5.2. Plaque Assays

Plaque assays were conducted on confluent L20B cell monolayers in 10 cm style petri dishes [33]. Petri dishes were challenged with 2 mL of concentrated sewage. The number of plaque forming units per ml (PFU/mL) was obtained by overlaying infected cell monolayers with a 1:1 mix of 2-fold concentrated M199 Medium (2-fold concentrated M199 medium, 2% FBS (*v*/*v*), and 1 mL (*v*/*v*) PSMY per 200 mL of medium) and a solution of 1.8% DIFCO Bacto Agar in water melted in a microwave. Plaques were visualized by addition of plaque staining solution (M199 medium, 1 mL per 100 mL 1% neutral red in H2O (*v*/*v*)). In some experiments, plugs of agar above all plaques or a subset of plaques chosen at random were transferred and amplified in L20B tube cultures, and the serotype of plaque purified poliovirus was determined using qRT-PCR (below) or by immunofluoresence assay (IFA).

#### 2.5.3. Immunofluorescence Assay (IFA) for Differentiation of Serotypes of Polioviruses

Immune fluorescence assays modified from [36] were conducted in 96-well tissue culture plates. L20B cell suspensions in 100 µL of complete viral growth medium (Eagle’s MEM-NAA 302 medium, 2% FBS (*v*/*v*), 4 mL per 500 mL PSMY) were added to each well. Ten-fold serial dilutions of virus suspension and positive controls (stock aliquots of each of the three Sabin strains) were prepared in the same medium. The plate was incubated at 37 °C overnight in a CO_2_ incubator after adding 25 µL of each dilution to 6 replicate wells. Replicate wells of the dilution that produced 25% CPE were identified by microscopy for immunofluorescence staining. Medium was removed from these and all other wells, and the cells were fixed for 10 min with 2 drops of a solution of 80% acetone and 20% PBS. After drying the plates, one drop of each mouse antibody was added to one replicate well (MerckMillipore ready-to-use; pan-enterovirus antibody; pan-polio antibody, anti-PV1, anti-PV2, and anti-PV3 antibodies), and the plate was incubated for 30 min at 37 °C. Liquid was removed, wells were washed twice with Tween20-PBS, one drop of anti-mouse IgG/FITC was added, and the plates were then incubated for 30 min at 37 °C. Then, liquid was removed, wells were washed twice with Tween20-PBS, and wells were then dried and inspected for specific fluorescence using an inverted fluorescence microscope.

#### 2.5.4. Molecular Assays

Viral RNA was usually extracted from 1 mL of tissue culture supernatants, stool suspensions, or concentrated sewage using a NucliSENS easyMag semiautomatic extractor (bioMérieux, Marcy l’Etoile, France) and the Specific B extraction protocol with easyMag extraction kits or their equivalent. RNA was extracted from some of the stool samples using a King Fisher (Thermo Scientific™, Waltham, MA, USA) semiautomatic extractor and the King Fisher System extractor RNA extraction kits according to manufacturer’s instructions. All extracted RNA samples were spiked with MS2 RNA. An RT-PCR result for MS2 that was >2-fold lower than MS2-RNA alone was considered to potentially contain PCR inhibitors, and RNA was either re-extracted and/or the sample was diluted and retested.

The serotype of the poliovirus in plaques, stool suspensions, supernatants of CPE positive tube cultures, or RNA extracted directly from concentrated sewage was determined by qRT-PCR using either the ITD v4.0 intratypic differentiation kits (CDC, Atlanta, GA, USA) or the in-house versions of these kits as well as qRT-PCR using primers specifically designed to detect WPV1-SoAS [37] (see Supplement Table S1 or Table S2 for primers and probes).

Direct quantitative RT-PCR (DqRT-PCR) for Sabin strains and WPV1-SoAS was conducted using the primers and the probes in Supplementary Table S1 or Table S2 and unamplified RNA extracted from weighed amounts of PV-positive stool suspended in a measured volume of PBS or from measured volumes of concentrated sewage where the initial volume and the final volume after concentration were measured. Analytic sensitivity was determined using serial dilutions of known concentrations of polioviruses as described in Hindiyeh et al. [37]. The analytic specificity of the WPV1-SoAS primers and probes was previously determined [37]. The analytic specificity of the Sabin primers and probes was ensured by using the same primer and probe sequences as in the WHO recommended poliovirus Intratypic Differentiation ITD 4.0 Kit. Plaque equivalent titers of virus in stool suspensions and in concentrated sewage were inferred after comparison to qRT-PCR results for serial dilutions of previously quantified poliovirus stocks.

### 2.6. Statistical Analysis

Continuous variables were examined for normal distribution using histogram and Q-Q plots. Continuous variables were expressed as mean plus standard deviations (SD) for normal distributions or median (interquartile range) for non-normal distributions. Categorical variables were described as frequencies and percentage. Plaque numbers were log transformed (base 10) to achieve normality. Linear regression was used to evaluate a population dilution factor constant, *CPop* (the dilution of virus excreted by an individual into the total amount of sewage generated by the catchment population). The equation for converting between Ct values and PFUs and the algorithm for inferring the number of excretors, *NExcr_PV-inf_*, were evaluated and met the assumptions for linear regression. The difference between observed numbers of individuals who were vaccinated and the estimated values, *NExcr_PV-est_*, using crude excretion rates (see above) was evaluated using the t-test (two-tailed with *p* < 0.05 considered as statistically significant). The correlation between log_10_ *NExcr_PV-inf_* and log_10_ *NExcr_PV-est_* from vaccination data was evaluated by Pearson product moment correlation or the Spearman’s rank correlation coefficient using SPSS Statistics for Windows v23 (IBM Corp, Armonk NY, USA). Agreement was assessed using the concordance correlation coefficient (CCC) in R v3.2.3 (R Foundation for Statistical Computing, Vienna, Austria). Bland and Altman plots with mean difference and ±1.96 SD were used to evaluate for fixed proportional biases. Demming regression was used to find calibration constant, *Cc*, in the calibrated algorithm (Equation (2)). All of the equations presented in our study are based on our statistical analyses.

### 2.7. Ethical Approval

The collection and the characterization of stools for poliovirus excretion during the asymptomatic outbreak of wild type 1 poliovirus in Israel during 2013–2014 were approved by the Institutional Review Board of Sheba Medical Center (SMC-0774-13) and exempted from a requirement to obtain informed consent. The number of individuals who were vaccinated with bOPV each week in the catchment population of each EnvS site during the asymptomatic outbreak of wild type 1 poliovirus in Israel in 2013–2014 was obtained from the National Vaccination Registry of Israel. Data from this study are ethically and legally restricted by the Institutional Review Board of Sheba Medical Center (contact person: Prof. Nati Keller, nati.keller@sheba.health.gov.il) and the Israel Center for Disease and Control and Prevention (contact person: Prof. Lital Keinan Boker, Director, lital.keinan2@moh.health.gov.il) to prevent compromise of patient identity. All links to personal details pertaining to or which could be used to identify individuals were removed. All data were analyzed anonymously.

## 3. Results

### 3.1. Comparison of Quantitative Tissue Culture-Based Plaque Assay Methods with Direct Quantitative RT-PCR (DqRT-PCR)

**Figure 1:**
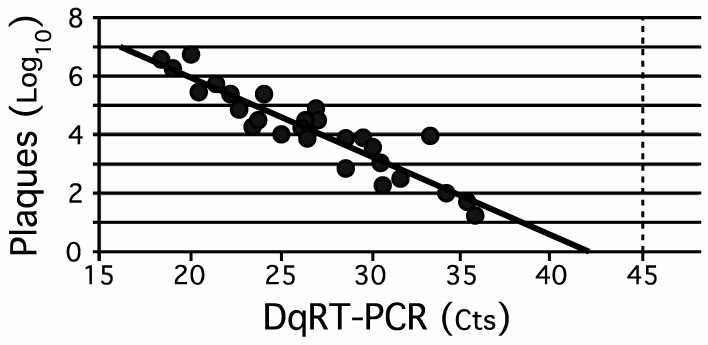
Comparison of the number of plaques (log_10_) and DqRT-PCR Ct values obtained for poliovirus RNA extracted directly from individual concentrated EnvS samples. The Log_10_ of the number of plaque-forming units (PFUs) of poliovirus in a concentrated sewage (Y-axis) was compared to the Ct values obtained by DqRT-PCR Ct poliovirus RNA extracted directly from the same concentrated sewage sample (X-axis). The dashed vertical line represents the final cycle of amplification.

Sewage concentrates (*n* = 55) that were WPV1-SoAS-positive by plaque assay and stored at −70 °C were thawed, and virus concentrations were re-determined in parallel by plaque assay [33] and DqRT-PCR [37]. The Ct value is the RT-PCR cycle at which a specific signal is first detected above threshold values. The Ct is inversely proportionate to the initial concentration of RNA being tested. Plaque numbers were log transformed (base 10) to achieve normality. Plaque numbers (log_10_) were graphed versus the Ct values from DqRT-PCR (Figure 1). Linear regression analysis of the quantitative results indicated a high degree of correlation between the two methods over an extended, 10^6^-fold range of concentrations (R^2^ = 0.85). Based on the antilog of the linear regression line presented in Figure 1, the measured results of one assay could be used to infer the expected result for the other as follows: *RTiter (in plaque equivalents)* = 10^(11.379—0.27 * *Ct*).^

### 3.2. Spiking/Recovery of OPV Serotypes from a Sewage System

Excretion by a single infected individual or close family contacts living at the same location was modeled by spiking a single EnvS site with aliquots of one or more monovalent vaccine strains. Excretion of polioviruses by two to three non-cohabiting individuals in a catchment area was modeled by simultaneously spiking two to three EnvS sites, each with a different monovalent vaccine strain. Samples taken at timed intervals from each downstream EnvS site were either pooled (composite samples) or kept as separate aliquots for quantification of recovered polioviruses. Two spiking/recovery protocols were tested.

In the first protocol, monovalent poliovirus vaccine stocks were rapidly poured into the sewage, and 125 mL EnvS samples were collected at multiple downstream EnvS sites at hourly intervals over two successive 24 h intervals. No polioviruses were recovered from any of the samples from any of the downstream EnvS site using this protocol.

In the second protocol, to ensure that spiked polioviruses would be harvested during the time spiked vaccine viruses traveled past the collection point, the interval between sampling was shortened to 6 or 10 min, and the spike for one of the three monovalent serotypes was slowly introduced into the sewage system over a 30 min interval using a peristaltic pump. The flow diagrams for two spiking experiments and the environmental surveillance sites used for recovery are presented in Supplement Figure S1 or Figure S2. Any longitudinal spreading of the virus spike through mixing and delays as the volume of sewage containing the spike traveled downstream would result in recovery of OPV virus-positive samples spread over an interval longer than 30 min.

**Figure 2:**
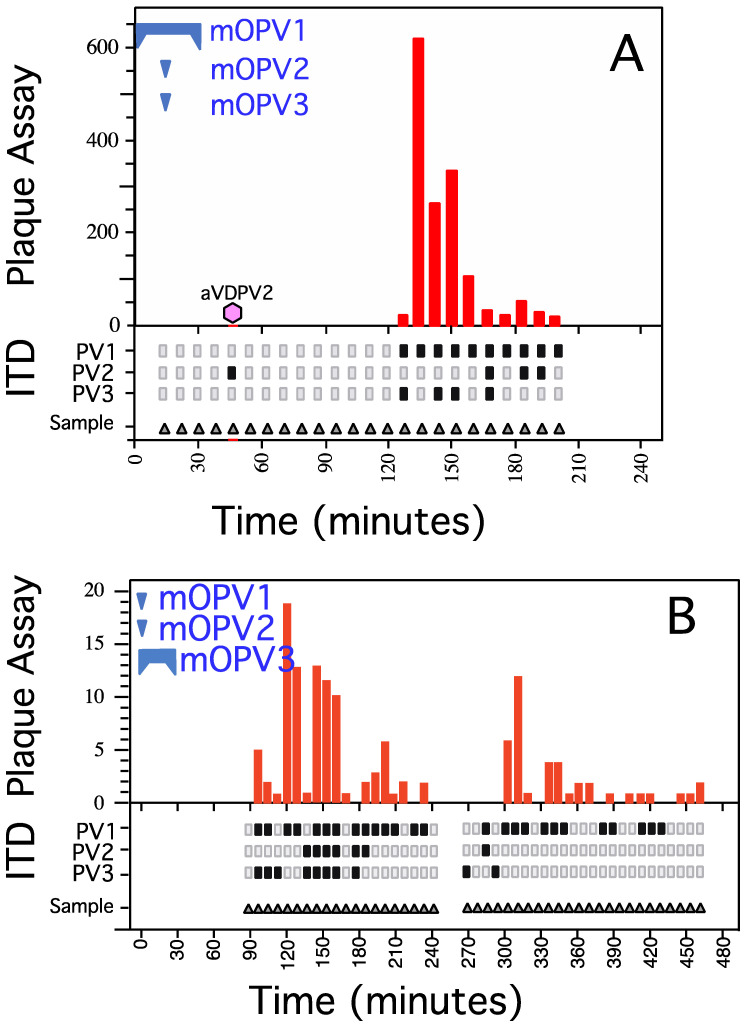
Addition of monovalent OPV serotypes to a sewer system and recovery of the spiked serotypes from sewage collected at EnvS site C75. Each panel in Figure 2 represents data from a separate sewage system poliovirus spike/recovery experiment. The flow diagrams for each complete spike/recovery experiment is in Supplementary Figure S1 or Figure S2. The poliovirus monovalent stocks used in the spikes and the times when they were introduced into the sewage system are indicated by blue downward pointing triangles or bars with downward pointing arrows at the top left of panels (**A**,**B**). The X-axis in each panel indicates the time in minutes after start of the peristaltic pump. The numbers in the Y-axis in the upper part of each panel represent the number of plaques recovered from EnvS samples. The red bars in the upper section of both panels indicate the number of plaques of confirmed poliovirus recovered at each individual harvest time-point. Intratypic differentiation results for these poliovirus plaques and polioviruses isolated in tube cultures from each sample are presented in the lower portion of each panel in separate rows of small rectangles, one for each serotype (PV1, PV2, and PV3). Dark black bars indicate that that serotype of poliovirus was identified by either ITD assay or IFA, while a light grey rectangle indicates that the given serotype was absent. The X-axis indicates the number of minutes after the start of the peristaltic pump. Upward pointing triangles immediately above the X-axis of each panel represent the times that each sample was harvested. The hexagon in panel (**A**) represents a single plaque of a highly diverged aVDPV2 recovered from a persistently infected individual in the catchment population. (Panel (**A**): Experiment 1) Spike: mOPV1 (4 × 10^10^ PFU) was introduced into manhole C121 over a 30 min interval; 15 min after the peristaltic pump was activated, mOPV3 (4 × 10^10^ PFU) was rapidly added into the same manhole, and mOPV2 (4 × 10^9 PFU^) was poured into an adjacent public flush toilet that emptied into C12, and the toilet was flushed twice. Recovery: Individual, un-pooled EnvS aliquots were collected at 6 min intervals from site C75. The serotype of polioviruses in poliovirus-positive tube cultures was determined by qRT-PCR using serotype-specific primers and probes of the ITD v4.0 intratypic differentiation kits (CDC, Atlanta GA) or the in-house versions of these kits. The serotype of poliovirus in representative plaques was determined by IFA. (Panel (**B**): Experiment 2) Spike: mOPV3 (4 × 10^10^ PFU) was introduced into manhole C121 over a 30 min interval. When the peristaltic pump was activated, mOPV2 (8 × 10^9^ PFU) was added to the same toilet as in panel (**A**)**,** and mOPV1 (4 × 10^10^ PFU) was added to a second public flush toilet at a remote site where effluent entered the sewage trunk line at a point downstream of C109 but upstream of C75. Both toilets were flushed twice. Recovery: Individual, un-pooled EnvS aliquots were collected at 6 min intervals from site C75. The serotypes of polioviruses in poliovirus-positive tube cultures were determined by qRT-PCR using serotype-specific primers and probes of the ITD v4.0 intratypic differentiation kits (CDC, Atlanta GA) or the in-house versions of these kits.

In the first experiment with the second protocol (Figure 2, Panels A), mOPV1 (4 × 10^10^ PFU) was introduced into sewer manhole C121 over a 30 min interval, mOPV3 (4 × 10^10^ PFU) was rapidly poured into the same manhole after 15 min, and mOPV2 (4 × 10^9^ PFU) was poured into a public flush toilet that was adjacent to and emptied into C121. The toilet was flushed twice 15 min after the peristaltic pump was activated. Sewage was collected at five downstream sites (Supplement Figure S1 or Figure S2). Three types of samples were collected: (1) pooled composite EnvS samples from aliquots collected at 10 min intervals at EnvS sites C109, C108, and Redding; (2) individual, un-pooled, EnvS aliquots collected at 6 min intervals from site C75; and (3) pooled EnvS samples from aliquots collected at hourly intervals over 24 h by an in-line composite sampler located at the entrance to the Shaf Dan sewage treatment plant. The EnvS samples were concentrated as described [33].

The number of polioviruses present in each concentrated sample was determined by plaque assay using L20B plate cultures. Plaque assay results for un-pooled aliquots recovered from the C-75 EnvS site are presented in the upper part of panel A of Figure 2. The serotype of polioviruses of representative plaques was determined by immunofluorescence assay. Polioviruses were recovered from C109 (138 plaques), C108 (78 plaques), and C75 (1368 plaques, Figure 2, Panel A) but not from the Redding or the Shaf Dan EnvS sites. The presence of recovered poliovirus in concentrated sewage was also determined by challenge of L20B tube cultures. The serotypes of polioviruses in the tube cultures were determined by qRT-PCR using serotype-specific primers and probes (see methods; primers are listed in Supplementary Table S1 or Table S2). 

The interval during which serotype specific OPV strains were recovered from un-pooled samples harvested from EnvS site C-75 is shown in the bottom section of Figure 2, panel A. Specifically, isolates of OPV1 (introduced over a 30 min period by peristaltic pump) were recovered at C75 over a 54 min interval, while OPV2 flushed from the toilet adjacent to C121 and OPV3 rapidly poured into C121 were recovered over 18 min intervals. Thus, the segment of sewage that contained the spike lengthened as it traveled downstream. Sequence analysis of a plaque represented by the hexagon in panel A revealed it to be a type 2 VDPV that diverged from type 2 OPV by 16% and which was genetically related to previously isolated, highly diverged, neurovirulent VDPV2s [20,22].

In a subsequent experiment using the second protocol (Figure 2, Panel B), mOPV3 (4 × 10^10^ PFU) was added by peristaltic pump over a 30 min interval at C121, mOPV2 (8 × 10^9^ PFU) was flushed from the same toilet adjacent to C121 as in the first experiment, and mOPV1 (4 × 10^10^ PFU) was flushed from a second public flush toilet at a remote site where effluent entered the sewage trunk line at a point downstream of C109 and upstream of C75 (see map in Supplement Figure S1 or Figure S2). Individual, un-pooled samples were collected at 6 min intervals as before at site C75 and by composite sampling at C109, C58, Redding, and at the entrance to the Shaf Dan sewage treatment plant. The presence of poliovirus in sewage concentrates was ascertained as before by challenge of L20B tube culture and by plaque assay on L20B cell culture monolayers. The number of poliovirus plaques that were recovered in each sample harvested from Shaf Dan-Br-C75 is shown in the upper portion of Figure 2, panel B. The serotype of polioviruses in each tube culture was determined by qRT-PCR using serotype-specific primers and probes (lower portion of Figure 2, panel B). OPV1 was absent from site C-109, as expected. OPV1 was recovered from EnvS sites C109, C75, C58, and Redding, but OPV1 was not present in the sample from the entrance to the Shaf Dan sewage treatment plant. OPV2 was not recovered from C109 or the entrance to the Shaf Dan sewage treatment plant but was recovered from EnvS sites C75, C58, and Redding. OPV3 was recovered from all sites except C58. At EnvS site C75, OPV1 was recovered over a 342 min interval, OPV2 was collected over a 54 min interval, and OPV3 was recovered over a 156 min interval, again indicating a limited longitudinal spread of virus as it traveled downstream.

### 3.3. Inferring the Number of Infected Individuals in a Catchment Area from Quantitative Recovery of Virus from Sewage

The number of poliovirus excretors, *NExcr_PV_*, in a catchment area is equal to the titer of virus recovered in the EnvS sample, *RTiter,* multiplied by the reciprocal of the average number of grams of stool excreted by an individual in a day (99.8 g, see discussion) multiplied by the average amount of virus excreted per gram of stool, *VEx_PV_*, and multiplied by the reciprocal of a crude population-based dilution ratio, *cDR*, that takes into account dilution of excreted poliovirus from a single individual by sewage generated by all of the non-infected individuals in the catchment population, *CPop*. This relationship is represented by Equation (1).

(1) $$NExcr_{PV}=\left\{\frac{RTiter}{\left(99.8\;VEx_{PV}\right)\left(cDR\right)}\right\}$$

For our calculations using Equation (1), values for the variable, *RTiter,* were in plaques measured directly by plaque assay or plaque equivalents calculated from Cts from DqRT-PCR assays. By the middle of a prolonged widespread poliovirus outbreak, infections no longer occur in synchronized waves. Thus, any given poliovirus-positive stool was possibly excreted during the beginning, the middle, or the end of an asymptomatic infection.

*VEx_PV_* for OPV1 was measured experimentally from 40 archived OPV1-positive stools that were collected from asymptomatic shedders after initiation of supplementary immunization with bOPV in 2013 [31]. *VEx_PV_* for WPV1-SoAS was measured experimentally from 47 archived WPV1-SoAS-positive stools that were collected from asymptomatic shedders during the outbreak of WPV1-SoAS in Israel in 2013–2014 [31]. We previously demonstrated that immunized individuals excreted poliovirus for up to 4 weeks after receiving a dose of OPV [34]. Assuming a similar time frame for WPV1-SoAS, the *Vex_PV_* corresponded to the average titer of poliovirus excreted per gram of stool by individuals who were infected with poliovirus within the previous four weeks. *VEx_PV_* was 2 × 10^6^ PFU/g of stool for WPV1-SoAS and 3 × 10^4^ PFU/g of stool for OPV1. These PFU equivalents were calculated from DqRT-PCR Ct values for RNA extracted from weighed amounts of PV-positive stool suspended in a measured volume of PBS. This was equivalent to 1.0 × 10^6^ PFU and 2.0 × 10^8^ PFU excreted per day for individuals infected with OPV1 and WPV1-SoAS, respectively.

The values for *CPop*, the catchment populations for each EnvS site, were obtained after consulting with the Bureau of Statistics and the Engineering Department in charge of sewage system treated by the Shaf Dan sewage treatment facility.

The population dilution modifier, *cDR*, was determined empirically from the spiking/recovery experiments described in the section above by linear regression analysis of the correlation between the catchment populations at sites C109, C108, C75, and Redding (10,000, 25,000, 80,000 and 800,000, respectively) and the plaque equivalent titer of mOPV1 recovered from those sites in the experiment described in part in Figure 2, panel B. The formula for the *cDR* regression curve was 0.187 plus (42,262 times the reciprocal of *CPop*) for each EnvS site. The R^2^ value for this inverse regression curve was 0.967.

### 3.4. Validating the Algorithm for Inferring the Number of Individuals Infected with OPV1

The number of individuals immunized with OPV1 who actively excreted OPV1 in a given catchment population during the supplementary immunization with bOPV, *NExcr_OPV1-inf_*, was inferred from the Ct values from DqRT-PCR of OPV1 RNA extracted directly from concentrated OPV1-positive EnvS samples by entering the value for *cDR* and the measured values for *VEx_OPV1_*, *RTiter,* and *CPop* into the algorithm (Equation (1)). In parallel, the actual number of individuals in the catchment population who were immunized each week during the four weeks prior to collection of each EnvS sample was obtained from National Vaccination Registry records. The number of these individuals who actively excreted OPV1, *NExcr_OPV1-est,_* was estimated from crude weekly excretion rates for OPV1 observed for individuals who received a dose of tOPV after prior vaccination with IPV and tOPV [34] as described in the methods section.

**Figure 3:**
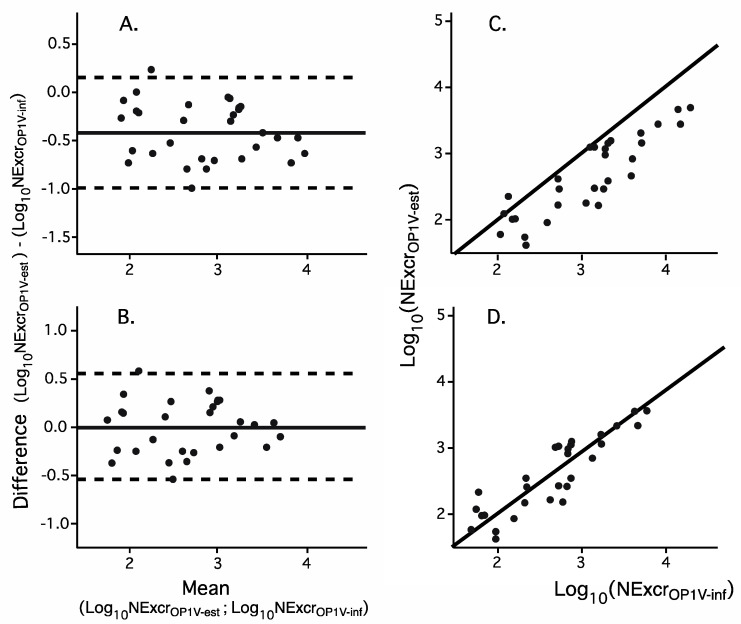
Comparison of (1) the number of excretors of OPV1, inferred by DqRT-PCR of OPV1 RNA extracted from EnvS samples (*Nexcr_OPV1-inf_*) and (2) the number of excretors estimated from vaccination records (*Nexcr_OPV1-est_*). Two methods were used to compare *NExcr_OPV1-inf_* with *NExcr_OPV1-est_.* Panel (**A**) represents the log_10_ of *NExcr_OPV1-est_* minus the log_10_ of *NExcr_OP1V-inf_* graphed against the mean of both log_10_ values. The dashed lines represent the 95% CI for the mean. Panel (**B**) represents a comparison of values of *NExcr_OP1V-inf_* for each EnvS catchment population calculated from DqRT-PCR with *NExcr_OPV1-est_* determined using vaccination records (X-axis and Y-axis, respectively). The solid black line in panel (**B**) represents the ideal curve if there was a one-to-one correlation between the log_10_ values estimated from the Vaccination Registry records and values inferred using the algorithm. *NExcr_OPV1-est_* and *NExcr_OP1V-inf_* were significantly correlated (two-tailed Students T test, *p* < 0.001). Panels (**C**,**D**) show the log_10_ *Nexcr_OPV1-inf_* values graphed in panels (**A**,**B**) were recalculated using the calibration constant in Equation (2) and re-graphed in panels (**C**,**D**), respectively.

We used two methods to compare *NExcr_OPV1-inf_* with *NExcr_OPV1-est_.* For the first comparison shown in Figure 3A, each dot represents the log_10_ of *NExcr_OPV1-est_* minus the log_10_ of *NExcr_OP1V-inf_* against the mean of both log_10_ values. The mean fell below zero, as did most of the calculated differences. The dashed lines represent the 95% CI for the mean. For the second comparison (Figure 3B), we graphed the values of *NExcr_OP1V-inf_* for each EnvS catchment population calculated from DqRT-PCR results against *NExcr_OPV1-est_* from vaccination records (X-axis and Y-axis, respectively). The solid black line in panel B represents the ideal curve if there was a one-to-one correlation between the log_10_ values estimated from the Vaccination Registry records and values inferred using the algorithm. The algorithm (Equation (1)) was validated by the good correlation between *NExcr_OPV1-est_* and *NExcr_OP1V-inf_* (two-tailed Students T test, *p* < 0.001).

### 3.5. Calibrating the Algorithm for Inferring the Number of Excretors of OPV1

We next calibrated the algorithm by calculating a constant, *Cc*, that would correct the *Nexcr_OPV1-inf_* values calculated from our algorithm so that the mean of the difference between estimated and inferred log_10_ values would be zero. The full algorithm modified with the *Cc* and including conversion from Cts to plaque equivalents is shown in Equation (2).

(2) $$NExcr_{OPV1-inf}=10^{-0.154+0.913\log_{10}\left\{\frac{\left(10^{11.379-0.27xCt}\right)}{\left(99.8\;VEX_{OPV1}\right)\left(0.187+\frac{42262}{CPop}\right)}\right\}}$$

The log_10_ *Nexcr_OPV1-inf_* values graphed in Figure 3, panels A and B, were recalculated using the calibration constant in Equation (2) and re-graphed in panels C and D, respectively. The resultant relations between the two values justify inclusion of the calibration factor in the equation.

### 3.6. Inferring the Number of Asymptomatic Excretors of WPV1-SoAS during the 2013–2014 Outbreak in Israel

During the asymptomatic WPV1-SoAS outbreak in Israel that started in 2013, plaque assays and DqRT-PCR molecular analysis of EnvS samples and stool surveys of asymptomatic children indicated that WPV1-SoAS circulated primarily among the Bedouin population in southern Israel [17,31]. The patterns of temporal changes in the number of WPV1-SoAS isolates recovered from sewage collected at five EnvS sites in southern Israel were determined by periodic re-sampling of each EnvS site. Catchment populations of EnvS sites at Arara, Arad-Br-Ksiefe, and Rahat wastewater treatment plants in southern Israel were primarily Bedouin, while catchment populations of EnvS sites at Beersheva and Ayalon-Br-Lod treatment plants included Jewish and non-Jewish families. Altogether, 16 EnvS samples were collected from Arara (from weeks 23 to 46); 13 from Arad-Br-Kseife (from weeks 28 to 46); 25 from Rahat (from weeks 11 to 46); 26 from Beersheva (from weeks 11 to 46); and 15 from Ayalon-Br-Lod (from weeks 28 to 46).

**Figure 4:**
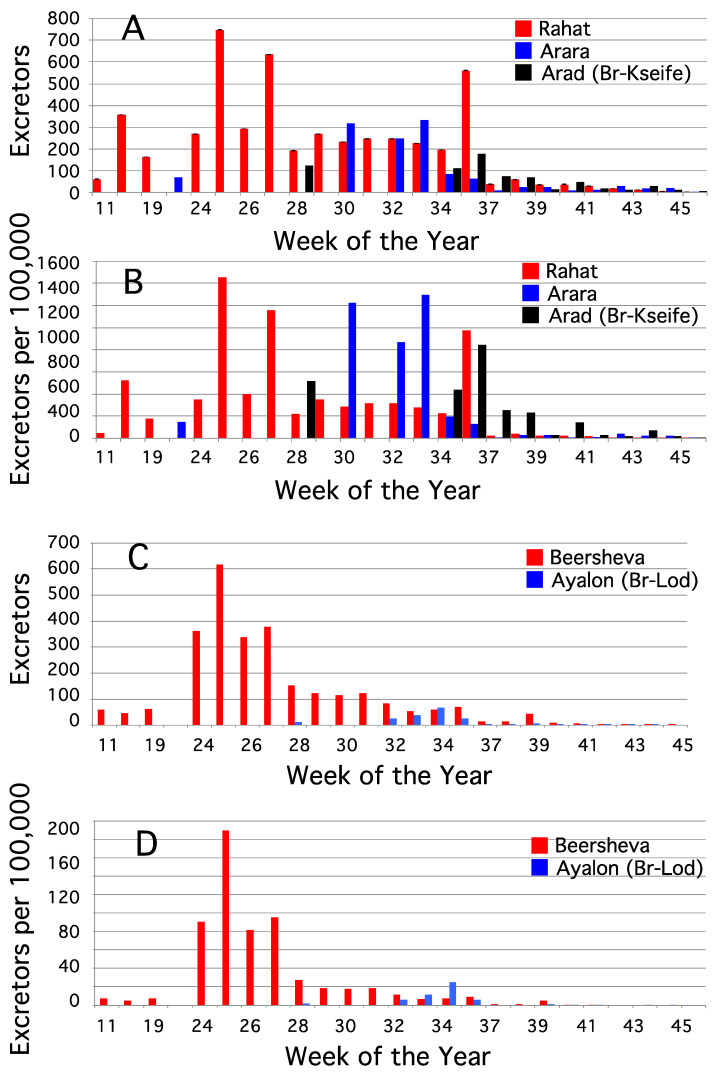
The inferred numbers of individuals who excreted WPV1-SoAS (*NExcr_WPV1-inf_*,) in five catchment populations in Israel during 2013 by the week the EnvS sample was obtained. In 2013, there was an asymptomatic outbreak of WPV1-SoAS. The total number of individuals who excreted WPV1-SoAS (*NExcr_WPV1-inf_*,) and the number of individuals who excreted WPV1-SoAS (*NExcr_WPV1-inf_*,) per 100,000 individuals were inferred for the catchment populations of Arara, Arad-Br-Kseife, and Rahat EnvS sites (panels (**A**,**B**), respectively). The total number of individuals who excreted WPV1-SoAS (*NExcr_WPV1-inf_*,) and the number of individuals who excreted WPV1-SoAS (*NExcr_WPV1-inf_*,) per 100,000 individuals were inferred for the catchment populations of Beersheva and Ayalon-Br-Lod EnvS sites (panels (**C**,**D**), respectively). The names of the EnvS sites indicate the names of the sewage treatment plants and should not be confused with specific cities or communities with the same name.

The number of WPV1-SoAS excretors, *NExcr_WPV1-inf_*, during 2013 and the number of excretors per 100,000 individuals in the catchment population were inferred for EnvS samples collected at Arara, Arad-Br-Kseife, and Rahat EnvS sites (Figure 4A,B, respectively) and for EnvS samples collected at Beersheva and Ayalon-Br-Lod (Figure 4C,D, respectively). *VEx_WPV1-SoAS-inf_* was substituted in Equation (2) for *VEx_OPV1-inf_*_,_ and *Rtiter* values were obtained either directly from the plaque assay results or after converting *Cts* from DqRT-PCR assays into plaque equivalents. As before, appropriate *CPop* values for each EnvS site were obtained after consulting with the Bureau of Statistics and the Engineering Department in charge of each of the five sewage systems. The number of excretors per 100,000 individuals inferred from our algorithm was highest in catchment populations of EnvS sites Arara, Arad-Br-Ksiefe, and Rahat, the catchment populations in southern Israel with the highest proportion of Bedouin children. Supplementary immunizations with bOPV were initiated on week 32 of 2013.

For the calculations, *VEx_WPV1-SoAS-inf_* was substituted in Equatio.n (2) for *VEx_OPV1-inf_*_,_ and *Rtiter* values were obtained either directly from the plaque assay results or after converting *Cts* from DqRT-PCR assays into plaque equivalents. *CPop* values for each EnvS site were obtained from the Bureau of Statistics and the Engineering Department in charge of each of the five sewage systems.

## 4. Discussion

The sustained transmission of WPV1-SoAS in a population with >90% three dose IPV vaccine coverage in Israel in 2013 in the absence of AFP cases stimulated development of an algorithm to infer the number of people in the catchment population of an EnvS site who were infected and excreted any WPV or circulating vaccine derived poliovirus in highly vaccinated populations. Knowledge gained before the outbreak was combined with data collected during the outbreak to develop and validate the model. The ability to accurately infer the number of infected individuals in a catchment population is critical for: (i) understanding the extent that the poliovirus already spread, (ii) making operational decisions such as expanding or reducing the number of surveillance sites and frequency of sampling, (iii) planning the type of intervention including vaccination policy, (iv) monitoring the effectiveness of intervention, and (v) demonstrating that WPV or circulating VDPV is no longer circulating in that or other catchment populations. The first step was to develop and evaluate methods for quantifying the amount of poliovirus in an EnvS sample when the virus of interest was present alone or together with vaccine strains. The second step was to quantify factors that affected recovery of poliovirus from EnvS samples. The third step was to determine the relative impact of the different factors on the amount of poliovirus that could be recovered from an EnvS sample. The final step was to develop, evaluate, and validate a model that would allow inferring the number of excretors from quantitative poliovirus assays of EnvS samples.

### 4.1. Step 1. Comparison of Quantitative Assays for Poliovirus

Plaque assays measure the titer of viable polioviruses in a suspension. The titer of poliovirus in an EnvS sample in plaques per ml is the number of plaques in a dilution of the sample that yields a countable number of non-overlapping plaques multiplied by the reciprocal of the dilution and by the un-concentrated volume divided by the volume of the concentrate. The plaque assay is excellent for quantification of infectious poliovirus, but it is technically difficult to use to determine the titers of a specific type of poliovirus in a mixture of polioviruses. This difficulty increases when WPV or VDPV of interest is not the major component in a mixture and especially when the poliovirus of interest is present in an excess of an OPV strain of the same serotype. High plaque numbers may occur in countries using OPV in routine immunization and after introduction of OPV strains in response to an outbreak in a country that exclusively vaccinated with IPV. In this situation, it is very labor intensive and time consuming to routinely serotype and characterize the virus in each plaque as vaccine-like, vaccine-derived, or wild when there are many plaques (intratypic differentiation). When polioviruses of interest are present at an average titer of one infectious virus per volume of aliquot tested, replicate aliquots may contain zero, one, or a few polioviruses. An example of this can be seen in Figure 2, where there where one to two plaques were isolated from an EnvS sample when tube cultures were negative or when tube cultures were positive and there were no plaques. This situation may also occur at the beginning and the end of poliovirus outbreaks and may require the testing of a sufficient number of replicate samples in parallel in order to obtain a representative picture of the composition of the original sample ([15,19,20,38] and this report).

Many polio laboratories in the global poliovirus laboratory network conduct World Health Organization recommended, quantitative RT-PCR assays to identify and conduct intratypic differentiation of poliovirus after amplification in tissue culture using qRT-PCR [39,40,41,42]. However, the results are qualitative even though a quantitative RT-PCR assay is used, since the amount of virus progeny in a tube culture where all cells are infected can come from a few cycles of replication when initial titers are high or from many cycles of replication when initial titers are low. DqRT-PCR, on the other hand, is quantitative because it uses specific primers and probes for the poliovirus of interest in the absence of the cell culture step and tests unamplified RNA extracted directly from concentrated sewage [37]. Specifically, in DqRT-PCR, the Ct is inversely proportional to the amount of specific poliovirus RNA extracted from the sample. A positive DqRT-PCR result implies that there was an active infection during which the poliovirus of interest was excreted. The disadvantage is that DqRT-PCR cannot indicate whether or how much of the specific RNA in the test sample comes from viable virus. The advantages of using DqRT-PCR over plaque assays and intratypic differentiation are that (i) it is more easily adapted to high throughput automation, (ii) it is less labor intensive than performing cell culture and molecular assays, and (iii) it decreases the turnaround time for initial identification of the polioviruses in the EnvS sample from two or three weeks to one week from sample collection, and it enables a significant increase in the number of EnvS samples that can be tested in parallel [21]. Equally important, DqRT-PCR allows quantitation of both the poliovirus of interest and OPV strains of the same serotype in EnvS samples containing homotypic mixtures upon designing and validating specific non-cross-reactive primers and probes, as was done for WPV1-SoAS [37] and OPV1 during the 2013 WPV1 outbreak in Israel.

Plaque assay results indicate viable virus concentrations, while DqRT-PCR Ct values represent RNA from non-viable poliovirus as well as viable poliovirus. Nonetheless, a comparison between the two indicated that it was possible to infer the result for one after measuring the result for the other. The correlation between results from both methods was significant (R^2^ = 0.85), and a formula for converting one value into the other was generated from this data. Once the relationship between the two assays was determined empirically, it was important to continue to maintain the conditions under which the assays were performed because the ratio of viable viruses among all virus offspring may vary depending on many factors. Environmental factors include biological and physical interactions that can occur while in the environment and the dwell time of the poliovirus in the environment between entry and collection. Physical factors include particle and chemical compositions of the EnvS sample, temperature conditions, and transportation and storage conditions. Laboratory factors include choice of methods and tissue culture conditions. An example illustrating the importance of being able to infer one quantitative result from the other occurred at the start of the vaccination campaign with bOPV in response to the 2013 WPV1 outbreak. Plaque assay results were used to follow weekly changes in relative environmental viral loads of WPV1 in EnvS samples prior to introduction of bOPV when WPV1 was the only replication competent poliovirus in the EnvS samples. After introduction of bOPV during the mass vaccination campaign, plaque assays were rendered impractical since >80% of EnvS samples contained high titers of vaccine strains. In contrast to plaque assays, the presence of the OPV1 polioviruses did not interfere with DqRT-PCR for WPV1. Converting Cts from DqRT-PCR into plaque equivalents enabled us to continue to express viral loads as plaque equivalents per ml of sewage.

### 4.2. Step 2. Identifying Measurable Factors That Impact on the Amount of Poliovirus That Can Be Recovered from an EnvS Sample

The efficiency of recovery of poliovirus after concentration of an EnvS sample can be determined in the laboratory relatively easily by spiking poliovirus-free EnvS samples with a known amount of poliovirus and then measuring the amount recovered in the concentrate. 

The proportion of poliovirus excreted by an individual in a catchment area population that ends up in the EnvS sample can also be modeled by spiking/recovery experiments. This process sounds relatively simple, however, in practice, it is very difficult. Adding poliovirus at one or more sites and collecting EnvS samples at one or more downstream sites must be carried out under field conditions that involve all aspects of the EnvS protocol and require coordination between laboratory personnel and sanitary engineers. Recovery is influenced by the amount of sewage that dilutes the spike by the time the spike reaches downstream collection sites and by the interaction of many environmental factors as the virus travels along the way to the collection sites [19,38,39,43]. Chemical and other contaminants present in sewage may inactivate polioviruses and may negatively affect cell culture or co-purify with poliovirus RNA and interfere with RT-PCR reactions. The distance between the spike site and the collection sites and the flow rate of the sewage between them determines the degree to which the spike is dispersed in the rest of the sewage by the time it flows past the collection point. Finally, ethical considerations dictate that addition of poliovirus to a public sewage system be as safe as possible and that polioviruses capable of replication and transmission are not re-introduced into a poliovirus-free area.

### 4.3. Step 3. Determining the Impact of Measurable Environmental Factors on Recovery of Polioviruses from EnvS Samples

The average percent recovery of poliovirus from EnvS samples in spiking recovery experiments using the Israel Sewage Surveillance Protocol, ISSP [31], was 8.5% (range 5.9–15.0%). These values fell within the range of our previous findings for recovery, which was 9% (range 5–14%) [44]. Sewage spiking experiments for recovery of poliovirus at EnvS sites downstream of the spike site were conducted using separate high titer, bulk monovalent stocks of monovalent OPV1, OPV2, and OPV3. The safety of each stock was confirmed as described in the methods section. The high titer enabled us to introduce aliquots directly into the sewage system without requiring any prior tissue culture amplification that might have resulted in the presence of some offspring that lost their attenuation to neurovirulence. An additional advantage of starting with monovalent stocks rather than using trivalent OPV is that, in a single experiment, different serotypes could be added at different locations or under different conditions, and available DqRT-PCR assays could independently measure the amounts of each poliovirus serotype in each EnvS sample collected downstream. Addition of two or more monovalent vaccine stocks at a single introduction site would be equivalent to excretion by a single infected individual or family, while the simultaneous introduction of individual stocks of monovalent vaccine at two to three different branch sites would be equivalent to excretion of poliovirus by more than one infected individual or family in a catchment area.

The sewage system feeding the Shaf Dan wastewater treatment plant (Supplement Figure S1 or Figure S2) was selected as an ideal location for a spike/recovery model for three reasons: (i) EnvS samples were analyzed more or less monthly since 1989; (ii) EnvS samples from the sewage system remained free of OPV strains from the beginning of 2005 after use of OPV was discontinued in Israel [18,20], thus the only source for OPV strains recovered during the experiments was from the spike that was added upstream of collection sites; and (iii) ethical considerations were not technically breached since highly diverged neurovirulent aVDPV2s were periodically isolated from sewage in the greater Tel Aviv region since 1998 [20,22], thus the spike would not be reintroduced into a poliovirus-free region.

In contrast to a spiking experiment reported by Hovi et al. [45] where poliovirus was recovered at a downstream site over a 4 day interval, no poliovirus was recovered at multiple downstream sites when sewage was collected at hourly intervals over 2 days in our initial experiments using protocol 1. It was important to readjust spiking conditions (protocol 2) so that enough poliovirus could be recovered in order to be able to quantitatively analyze experimental results. The pattern of poliovirus recovery (Figure 2) indicated that the temporal spread for virus flowing past collection sites was increased to only a few hours for the central Israel sewage system as compared to the multi-day temporal spread observed by Hovi et al. [45]. A longer path, a slower flow rate, and/or a difference in the manner in which the flush was diluted might explain the greater temporal dispersion of the spike from toilet 2 compared to toilet 1. Sanitation engineers ruled out an alternate explanation by indicating that there was no holding tank or holding area between toilet 2 and trunk line C that could significantly delay portions of the spike.

### 4.4. Step 4. The Development, Evaluation, and Validation of a Model for Inferring the Number of Excretors from Direct EnvS Quantitative Poliovirus Assay Results

There are a number of ways to model the dilution of poliovirus in excreta from an infected individual as it travels downstream in a sewage system that relate to the catchment population. For the algorithm in this report, the uninfected population dilution factor was calculated empirically using regression analysis of the amount of a single spike that was recovered at multiple downstream sites with known successively increasing catchment populations. Alternative methods take into account average water usage per person multiplied by the number of individuals in the catchment area or the total amount of sewage that flows through the inlet of the sewage treatment plant over 24 h multiplied by the ratio of the catchment population of the EnvS site to the entire catchment population of the sewage treatment plant.

Measuring the concentration of poliovirus per gram of stools is relatively straightforward but may be affected by a non-homogenous distribution of the virus in the stool. The average amount of poliovirus per gram of stool was determined for WPV1-SoAS and OPV1 using convenient poliovirus-positive stool samples collected before and after the bOPV response to the WPV1 outbreak in 2013, respectively [21,31]. Infections with both polioviruses at the time of stool collection were unsynchronized. Specifically, WPV1-SOAS-positive stool samples were taken at a time when the amount of virus in sewage remained at a prolonged peak for at least 3 months [21], and dates of bOPV vaccination obtained from the national vaccination registry indicated that OPV1-positive stools were collected from individuals who were vaccinated at varying intervals before collection. Excretion of 10^2^-fold more WPV1-SOAS per gram of stool than OPV1 is consistent with similar differences between different poliovirus strains observed by Lodder et al. [46]. An alternative explanation for the difference may be that up to 59% of the children were possibly asymptomatically infected with WPV1-SoAS [47], and this partially depressed the replication of OPV1.

It was less straightforward to choose a value for the average mass of stools excreted per day by a single infected individual for calculating the total amount of poliovirus excreted per day [15,19,38,46]. Based on literature reports, the average daily mass of excreta ranged between 35 and 500 g: (35 to 255 gm per day [48], 89.7 to 149.2 gm per day over 30 days [32], 100 gm per day [49], 100–200 gm per day for healthy adults [50], <150 gm per day in young infants [51], 100 g to 500 gm per day [46], and ≤200 gm for adults in western countries [52]. An average intermediate value of 99.8 gm of feces corresponding to the mass excreted per day over a 30 day period for adults following a controlled diet [32] was chosen for the algorithm.

The next step toward inferring the number of infected individuals from quantitative measurement of poliovirus in EnvS samples was to relate the average amount of virus excreted per day by an infected individual to the poliovirus recovered from a known number of infected individuals in the catchment population. Lodder et al. [46] observed that poliovirus concentrations in sewage approximated the amount of poliovirus initially excreted in stools after determining poliovirus concentrations by plaque assay in stools for up to 56 days and EnvS samples daily for 9 days and afterwards intermittently for up to 62 days after elderly naïve, vaccinated, or naturally exposed individuals received a single dose of mOPV1 or mOPV3. The study described here differed from that of Lodder et al. [46] in a number of aspects. OPV1 was excreted primarily by children under 10 years of age who received a dose of bOPV only if they previously received one IPV dose (>90% actually received three doses) and not adults [15,17,31,53]. Vaccination dates were retrospectively retrieved for a single convenient stool sample from each individual in contrast to studying longitudinal excretion by individuals. DqRT-PCR allowed easy quantification of OPV1. The numbers of individuals in the catchment population who were immunized within one to four weeks prior to collection of the EnvS sample was accurately retrieved from the National Vaccination Registry. Finally, we estimated the number of vaccinated individuals who would actively excrete OPV1 who were vaccinated in the 4 weeks prior to collection of the EnvS sample based on crude excretion rates for children exposed to a dose of OPV after receiving three doses of IPV and two or three doses of OPV [34]. This last approximation possibly underestimated the number of excretors since it did not exactly model the situation during the 2013 outbreak where all individuals who received bOPV previously received IPV but only some were previously exposed to wild, infectious WPV1-SoAS poliovirus.

The number of individuals excreting OPV1 inferred from DqRT-PCR results using our algorithm was significantly correlated (*p* < 0.005) with the estimated numbers of individuals who were vaccinated with bOPV and who excreted OPV1 after receiving bOPV. The three-fold and the five-fold differences between 98.9 g of stool per day and the minimum and the maximum masses of excreted stools, respectively, influenced the number of excretors inferred from DqRT-PCR Cts to a much lesser extent in our algorithm than the 10^2^-fold difference in virus concentrations between strains reported here and also seen by Lodder et al. [46]. The constant difference between the log_10_ of the estimated numbers of vaccines who excreted OPV1 and the number of excretors inferred from our algorithm related to and corrected some of the approximations described above. A constant correction factor calculated to correct the mean of the difference to zero was therefore introduced into the algorithm for inferring the weekly number of individuals who excreted poliovirus.

In a final step, the algorithm with correction factor was used to infer the weekly number of individuals who excreted WPV1 in different catchment areas during the 2013 outbreak using DqRT-PCR results (Figure 3.). Bercenko et al. [15] used the same bOPV vaccination data and the DqRT-PCR data for WPV1 in the week before the initiation of immunization with bOPV to infer that 1663 individuals were infected with WPV1 in Rahat compared with 246 inferred by our algorithm for the same week. This approximately seven-fold difference may be due in part to the fact that we took into account the 10^2^-fold higher amount of WPV1 than OPV1 excreted per gram of stool in our algorithm, whereas Bercenko et al. did not, that our calculations were based on observations over a much longer period of the outbreak, and that some of the approximations based on observations of spiking/recovery experiments in the central sewage system might have differed if similar spiking/recovery experiments were carried out in the sewage system of Rahat. In Brouwer et al. [47], we used a deterministic, compartmental, susceptible, exposed, infectious, and recovered (SEIR) infectious disease model based on the DqRT-PCR environmental surveillance in Rahat to measure the epidemic curve and the transmission dynamics. The R0 was 1.62 (95%CI 1.04–2.02), and the model indicated that 59% (95%CI 9–77%) of individuals who did not receive OPV (mostly children under 10 years of age) were infected with WPV1-SoAS by the end of the outbreak.

Another confounding factor to consider when inferring the number of individuals infected with WPV1 or cVDPV is that poliovirus excreted by individuals during an outbreak results from infections after exposure to varying amounts of poliovirus from other individuals or the environment, whereas almost all but not all of the excreted OPV1 may come from primary infections after a single exposure to a constant high dose of OPV1. For example, two additional small peaks in poliovirus recovered from sewage on day 22 and possibly day 36 but not from stools of the vaccinated individuals in the experiment by Lodder et al. were attributed [46] to a limited number of secondary infections.

The relative logic applied in constructing the formulas is broadly applicable for the approach described here. However, the actual values depend on factors that differ between EnvS sites, catchment populations, and characteristics of the pathogen. When two different methods provide reproducible results that are correlated, it is possible to introduce a correction for unknown and/or unmeasured constant factors so that the mean of the difference between the two results approaches zero (see Section 3.3 and Section 3.4 above).

EnvS is invaluable for demonstrating that poliovirus transmission and poliovirus infections ceased at the end of an outbreak [19,39]. Confidence that poliovirus is truly absent in the catchment population of a specific EnvS site increases when assays of subsequent samples collected monthly for 6 to 12 months at that site remain negative and even more so when all other EnvS samples remain negative during this period. EnvS measures virus excreted by asymptomatic and symptomatic infections but does not measure individual clinical outcomes. Classically, it is viewed as a supplement. However, in certain situations, such as the WPV1-SoAS outbreak in Israel discussed here, we would only have noticed the outbreak when it was too late if we relied on detecting cases when they occurred. The effectiveness of EnvS for detecting circulation relative to symptom-based surveillance increases when vaccine coverage is high and paralytic infection rates are low. The main limitations of using EnvS exclusively include: (1) EnvS is most effective when performed on catchment populations with centralized sewage systems which do not exist in many parts of the world [14]; (2) negative test results do not distinguish between true absence of the target or presence at levels below the limits of detection [20]; and (3) increasing confidence that a negative finding represents a true absence of targets requires a history of previous negative findings at that site and additional sites, inclusion of appropriate QA and QC standards such as demonstrating the ability to detect non-polio enteroviruses, and inclusion of process controls (example MS2 RNA) in all samples and throughout the entire process from collection to analysis [15,20]. It is also important to validate the recovery/detection process for the pathogen. Methods that work for non-enveloped viruses, such as polioviruses, may not be as efficient or reproducible when applied for detecting enveloped viruses such as COVID-19.

## 5. Conclusions

In conclusion, we examined and compared quantitative methods for determining the titer of polioviruses in EnvS samples and validated the application of quantitative results toward inferring the number of individuals who excrete poliovirus during an outbreak and subsequent vaccination campaigns. Inferring the number of infected individuals is an important addition to our previous ability to provide information on weekly changes in the concentration of poliovirus recovered from sewage during an outbreak [15,17,21]. We validated our model using quantitative poliovirus information from EnvS samples taken during a real outbreak of WPV1 where no AFP cases occurred and from knowledge gained before the outbreak. Some of our observations are particular to the sewage network and the catchment population under study and should be determined empirically as outlined here. These include: characteristics of the sewage network such as water use per person, composition of waste water, and physical properties including infrastructure, temperature, volume, and flow rate of the sewage in the network; laboratory conditions such as recovery efficiency for the pathogen; assay and reagent differences; population differences such as the effect of different diets on the stool volume and the prior history of vaccination/exposure; and pathogen related differences such as strain-specific differences in the amount of virus excreted, the time during which virus is excreted, the basic reproduction number for the variant (R0), and the extent that a virus variant can escape immunological blocking if the host was previously exposed to a vaccine strain or a different variant. The methodology described here provided rapid epidemiological information on what was transpiring in the catchment populations of the EnvS sites that were sampled and by extrapolation in the entire population. DqRT-PCR is much less labor and resource intensive than other surveillance methodologies. It does require development and validation of specific reagents as soon as possible after identification and characterization of a poliovirus of interest [37]. 

An important legacy of our work is to apply quantitative EnvS methods to infer the number of excretors of other human enteric pathogens where there are: (1) significant numbers of asymptomatic infections; (2) long incubation times during which infectious virus is excreted; and (3) limited resources, facilities, and manpower that restrict the number of individuals who can be tested and re-tested. The methodology is effective whether or not effective replication competent vaccines are available for disease prevention since largescale, widespread individual clinical testing could be substituted for recovery of replication competent vaccine strains for validation and calibration. Our method could also be used to provide vital information during viral outbreaks where the main locus of infection may not be the enteric system but where the virus is still excreted in stools. For example, large amounts of coronavirus are excreted in stools during infections with coronaviruses such as SARS [54], MERS [55], and SARS-CoV-2 [56,57], the etiological agents for severe acute respiratory syndrome, Middle Eastern respiratory syndrome, and COVID-19, respectively, although they primarily infect the respiratory system and are spread by respiratory secretions. Quantitative environmental surveillance alone corelates with the number of infected individuals, and sequential monitoring of the same EnvS site correlates with dynamic changes in the number of infected individuals. However, without actually measuring the additional factors described here, it is difficult to compare quantitative results from different locations during outbreaks and pandemics and to infer the actual numbers of infected individuals. 

When expanding the use of quantitative wastewater-based epidemiology to other viruses such as SARS-CoV-2, it is necessary to determine the analytic uncertainty at each of the following stages: (1) virus shedding into sewers; (2) sample collection; (3) transportation and storage; (4) concentration; (5) quantitative analysis of the virus concentration in wastewater (including determinations of linearity of response, absolute limits of detection (LOD) and quantitation (LOQ), and inter-experiment repeatability over the dynamic range); and (6) normalization and interpretation, including inferring of numbers of infected individuals [58,59,60,61]. Standardized protocols, laboratory equipment, sample processing strategies, appropriate quality controls, methods for preparing standard curves, and performance limits need to be established for each new pathogen in order to enable inter-laboratory comparisons [62,63]. As for poliovirus surveillance [10], it is essential to develop explicit performance standards and proficiency testing panels to validate the methods selected to enhance the ability to compare findings between laboratories [64]. Limitations of quantitative WBE for SARS-CoV-2 are discussed in detail in McGonical et al. [61], and additional detailed info on survivability recovery and quantitative analysis of other water pathogens can be found in book chapters available online at The Global Water Pathogens Project (GWPP) Available on-line: https://www.waterpathogens.org/ accessed on 31 July 2021). It is important to stress that laboratory assays need to be optimized for environmental samples, not clinical samples [61], and to realize that surrogate spikes may partition differently in wastewater than in authentic in-situ SARS-CoV-2 [63]. At present, there is a lack of standardized detection and quantification methods for enveloped viruses [65]. Enveloped viruses, including SARS-CoV-2, tend to decay or be inactivated faster than non-enveloped viruses in most concentration methods that were initially designed for non-enveloped viruses [65,66]. Finally, viral fitness and the amount of virus excreted may change during outbreaks of newly emerging pathogens at different stages in the outbreak as different variants become dominant [67] and as duration of exposure and viral loads at exposure change in relation to interventional strategies (including WASH (water safety, sanitation, and hygiene), social distancing, and vaccination).

## Data Availability

Data from this study are ethically and legally restricted by the Institutional Review Board of Sheba Medical Center (Contact person: Prof. Nati Keller, nati.keller@sheba.health.gov.il), and the Israel Center for Disease and Control and Prevention (Contact persons: Prof. Lital Keinan Boker, Director, lital.keinan2@moh.health.gov.il) to prevent compromise of patient identity.

## Supplementary Materials

The following are available online at https://www.mdpi.com/article/10.3390/vaccines9080870/s1: (Note: The Supplementary Material is presented in two files. In the first, the map in Figure S1 is high definition, while in the second, the same map is presented as Figure S2 but in in lower definition.). 1. High-definition Supplementary Material File: Figure S1: Environmental Surveillance (EnvS) Sites in the Sewage System of Greater Tel Aviv, Israel, Used for Recovering Oral Poliovirus Vaccine Isolates Downstream of Sites Spiked with Monovalent Oral Poliovirus Vaccine Strains. Table S1: Primers and probes used for direct DqRT-PCR. 2. Lower-definition Supplementary Material: Figure S2: Environmental Surveillance (EnvS) Sites in the Sewage System of Greater Tel Aviv, Israel, Used for Recovering Oral Poliovirus Vaccine Isolates Downstream of Sites Spiked with Monovalent Oral Poliovirus Vaccine Strains. Table S2: Primers and probes used for direct DqRT-PCR.
