# Supplementary material for: Inferring Numbers of Wild Poliovirus Excretors Using Quantitative Environmental Surveillance
Source: Vaccines (Basel). 2021 Aug 6;9(8):870. doi: 10.3390/vaccines9080870 (PMC8402366; doi:10.3390/vaccines9080870)
Supplement: Supplementary file 1 [file vaccines-09-00870-s001.zip › SUP/vaccines-1259784 Supplement Hires.pdf]

Supplementary Materials for

Inferring Numbers of Wild Poliovirus Excretors using Quantitative Environmental Surveillance

Yuri Perepliotchikov[1]†, Tomer Ziv[2], Musa Hindiyeh[1,2]‡, Yossi Manor[1,] §, Danit Sofer[1], Jacob Moran-Gilad[3,4], Laura Stephens[5], Ella Mendelson[1,2], Merav Weil[1], Ravit Bassal[6], Emilia Anis[3,7], Shepherd Roee Singer[3,4], Ehud Kaliner[3], Gillian Cooper[5], Manasi Majumdar[5], Michal Markovitch[6,] ‖, Daniela Ram[1,] ¶, Itamar Grotto[3,4], Ronni Gamzu[2,3,] **, Javier Martin[5], Lester M. Shulman[1,2,] ††,*

*correspondence to Lester M. Shulman, Central Virology Laboratory, at Sheba Medical Center, Tel Hashomer, 52621, Israel. lester.shulman@sheba.health.gov.il

This PDF file includes:

Supplementary Figure S1 (High resolution)**.** Environmental Surveillance (EnvS) Sites in the Sewage System of Greater Tel Aviv, Israel, Used for Recovering Oral Poliovirus Vaccine Isolates Downstream of Sites Spiked with Monovalent Oral Poliovirus Vaccine Strains.

Supplement Table S1. Primers and probes used for direct DqRT-PCR.

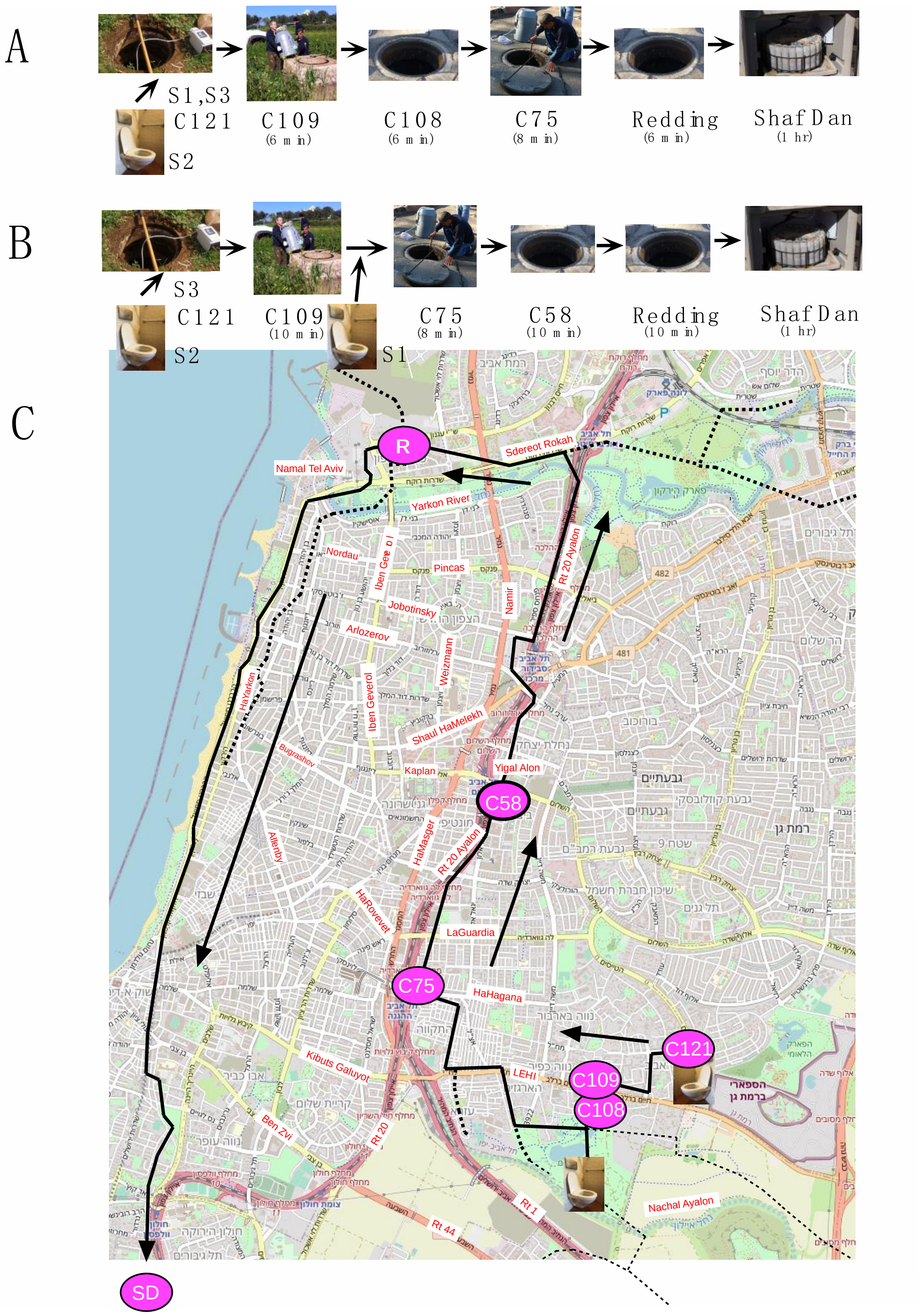
A
S1,S3
C121
S2
C109
(6 min)
C108
(6 min)
C75
(8 min)
Redding
(6 min)
Shaf Dan
(1 hr)
B
S3
C121
S2
C109
(10 min)
S1
C75
(8 min)
C58
(10 min)
Redding
(10 min)
Shaf Dan
(1 hr)
C
R
Namal Tel Aviv
Sdereot Rokah
Yarkon River
Rt 20 Ayalon
Nordau
Iben Gevirol
Pincas
Jobotinsky
Namir
Arlozerov
Weizmann
HaYarkon
Iben Geverol
Shaul HaMelekh
Bugrashov
Kaplan
Yigal Alon
C58
Allenby
HaMasger
Rt 20 Ayalon
HaRoveve
LaGuardia
C75
HaHagana
C121
Kibuts Galuyot
LEHI
C109
C108
Ben Zvi
Rt 20
Rt 1
Nachal Ayalon
Rt 44
SD

Figure S1. Environmental Surveillance (EnvS) Sites in the Sewage System of Greater Tel Aviv, Israel, Used for Recovering Oral Poliovirus Vaccine Isolates Downstream of Sites Spiked with Monovalent Oral Poliovirus Vaccine Strains.

Panels A and B: Flow chart for representative experiments using spiking-recovery Protocol 2. Aliquots of the three poliovirus serotypes were introduced separately at C121 and at one or more public toilets emptying into Branch C of the Shaf Dan sewage system (Panel C). The serotype introduced into the sewage system at site C121 was dripped into the site over a 30-minute period using a peristaltic pump, while toilets were flushed twice. The interval between collections of aliquots by the portable composite samplers was 6 or 10 minutes as indicated under the name of each EnvS site in Panels A and B. With this interval, a minimum of at least two to three samples would be expected to be collected at each downstream collection site that would potentially contain poliovirus from the spike introduced by the peristaltic pump. Batteries and collection bottles were swapped out with fresh bottles and batteries for back-to-back continuous collection runs. The portable automatic sampler at C75 was modified to collect individual, un-pooled samples at 6-minute intervals that were analyzed separately to provide a more detailed time course for recovery. A single 24-hour composite sample was obtained from the in-line automatic composite sampler located at the inlet into the Shaf Dan Wastewater Treatment Plant by pooling aliquots collected at hourly intervals.

Panel C: Map of the sewage system in the greater Tel Aviv area. The ShafDan Waste Water Treatment Plant and sewage system in central Israel are managed by Igudan, the Dan region Association for Environmental Infrastructure. The solid black line superimposed on the street map of the greater Tel Aviv area represents the branches and main trunk line running from the spiking sites depicted in Panels A and B to the ShafDan Waste Water Treatment Plant (oval at the bottom labeled “SD”) as they existed in 2011 when the Spiking-recovery experiments were performed. The direction of flow is indicted by the black arrows. Dashed lines indicate major side branches that joined into this path. EnvS sites relevant for this publication are indicated by an oval with the ID of the EnvS site within the oval. The wastewater flowed north by gravitation to the Reading pumping station. The wastewater was then pumped upwards at Reading and again at a point downstream so that it could flow southwards by gravitation to the Shaf Dan Waste Water Treatment Plant, Igudan, located south of Tel Aviv. (The section of the trunk line between the arrow at the lower righthand side of the map and the ShafDan Waste Water Treatment Plant is not shown.) EnvS samples were collected (“recovery”) by automatic, computerized composite samplers (Sigma SD900 portable samplers, HACH, Loveland, CO, United States) at sites indicated by the ovals and by an in-line composite sampler at the mouth of the Shaf Dan Waste Water Treatment Plant. Catchment populations based on the Bureau of Statistics and the Engineering Department in charge of sewage system treated by the Shaf Dan Sewage treatment facility at the time were 10,000 for C109; 25,000 for C108; 80,000 for C75, 800,000 for Redding, and 1,600,000 for the Shaf Dan Waste Water Treatment Plant.

(The public access street map of the greater Tel Aviv Area contains information from OpenStreetMap and OpenStreetMap Foundation, which is made available under the Open Database License. https://www.openstreetmap.org/#map=14/32.0762/34.7936. We acknowledge help from Yuval Sela, Head Engineer, Igudan, who provided pipeline maps used in the preparation of the overlay on the street map.)
An interactive map showing the current configuration of the sewage system in greater Tel Aviv can be accessed at https://www.google.com/maps/d/u/0/edit?mid=1l6DCWPDIbHxk84wWwQJWrQLzXKM&usp=sharing).

Supplement Table S1. Primers and Probes used for direct DqRT-PCR.

Primer/Probe	Sequence (5′ -> 3′)	Ref	Position[a]
Sabin 1 PCR-1 (A)	CCACTGGCTTCAGTGTTT	1	2600–2583
Sabin 1 PCR-2 (S)	AGGTCAGATGCTTGAAAGC	1	2505–2523
Sabin 1 Probe A4	CY5-CGCCCCCACCGTTTCACGGA-BHQ3	1	2559–2540
Sabin 2 PCR-1 (A)	CGGCTTTGTGTCAGGCA	1	2595–2579
Sabin 2 PCR-2 (S)	CCGTTGAAGGGATTACTAAA	1	2525–2544
Sabin 2 Probe	FAM-ATTGGTTCCCCCGACTTCCACCAAT-BHQ1	1	2550–2572
Sabin 3 PCR-1 (A)	TTAGTATCAGGTAAGCTATC	1	2591–2572
Sabin 3 PCR-2 (S)	AGGGCGCCCTAACTTT	1	2537–2552
Sabin 3 Probe	ROX-TCACTCCCGAAGCAACAG-BHQ2	1	2554–2571
WPV1-SoAS (S)	5′-TCATCCAGCACAGGTCACGA-3′	2	2678–2697
WPV1-SoAS Probe	5′-TACGTGATTTTCCACACTGA-3′	2	2816–2797
WPV1-SoAS (AS)	FAM-5′- AATGACTGTAGACAATTCCGCCT-‘3-BHQ2	2	2748–2770

[a]Position relative to the VP1 positions reported by Toyoda et al. for Sabin primers and probes or WPV1-SoAS (KJ019831). S=Sense, A=Antisense. Deoxyinosine residues are indicated by (I). Abbreviations for nucleotides follow the International Union of Biochemistry nomenclature: B, cytosine, guanine, or thymine; H, adenine, cytosine, or thymine; K, guanine or thymine; I, inosine; M, adenine, cytosine, N, adenine, cytosine, guanine, or thymine; R, adenine or guanine; Y, cytosine or thymine. FAM is 6-carboxyfluorescein and BHQ is a black hole quencher 1, 2, or 3.

References:

1. Poliovirus rRT-PCR ITD 4.0 Kit. A kit for the serotyping of L20B positive cell cultures and intratypic differentiation of polioviruses in support of the Global Polio Eradication Initiative. Distributed by The WHO Collaborating Centre for Enteroviruses and Polioviruses, Centers for Disease Control and Prevention, 1600 Clifton Road NE, Mailstop G-10, Atlanta, Georgia 30333 USA, +1-404-639-1341, Fax: +1-404-639-4011. Email: MMandelbaum@cdc.gov, HSun@cdc.gov, SOberste@cdc.gov.
   For research use only. Not for use in diagnostic procedures. November 20, 2014.

2. Hindiyeh MY, Moran-Gilad J, Manor Y, Ram D, Shulman LM, Sofer D, Mendelson E. Development and validation of a real time quantitative reverse transcription-polymerase chain reaction (qRT-PCR) assay for investigation of wild poliovirus type 1-South Asian (SOAS) strain reintroduced into Israel, 2013 to 2014. Euro Surveill **2014**; 19(7): 20710.
